# Increasing ATP turnover boosts productivity of 2,3-butanediol synthesis in *Escherichia coli*

**DOI:** 10.1186/s12934-021-01554-x

**Published:** 2021-03-09

**Authors:** Simon Boecker, Björn-Johannes Harder, Regina Kutscha, Stefan Pflügl, Steffen Klamt

**Affiliations:** 1 Max Planck Institute for Dynamics of Complex Technical Systems, Sandtorstraße 1, 39106 Magdeburg, Germany; 2 Institute for Chemical, Environmental and Bioscience Engineering, Research Area Biochemical Engineering, Technische Universität Wien, Gumpendorfer Straße 1a, 1060 Vienna, Austria

**Keywords:** *Escherichia coli*, Enforced ATP wasting, Biofuels, Two‐stage process, Butanediol, Productivity

## Abstract

**Background:**

The alcohol 2,3-butanediol (2,3-BDO) is an important chemical and an *Escherichia coli* producer strain was recently engineered for bio-based production of 2,3-BDO. However, further improvements are required for realistic applications.

**Results:**

Here we report that enforced ATP wasting, implemented by overexpressing the genes of the ATP-hydrolyzing F_1_-part of the ATPase, leads to significant increases of yield and especially of productivity of 2,3-BDO synthesis in an *E. coli* producer strain under various cultivation conditions. We studied aerobic and microaerobic conditions as well as growth-coupled and growth-decoupled production scenarios. In all these cases, the specific substrate uptake and 2,3-BDO synthesis rate (up to sixfold and tenfold higher, respectively) were markedly improved in the ATPase strain compared to a control strain. However, aerobic conditions generally enable higher productivities only with reduced 2,3-BDO yields while high product yields under microaerobic conditions are accompanied with low productivities. Based on these findings we finally designed and validated a three-stage process for optimal conversion of glucose to 2,3-BDO, which enables a high productivity in combination with relatively high yield. The ATPase strain showed again superior performance and finished the process twice as fast as the control strain and with higher 2,3-BDO yield.

**Conclusions:**

Our results demonstrate the high potential of enforced ATP wasting as a generic metabolic engineering strategy and we expect more applications to come in the future.

**Supplementary Information:**

The online version contains supplementary material available at 10.1186/s12934-021-01554-x.

## Introduction

**Fig. 1:**
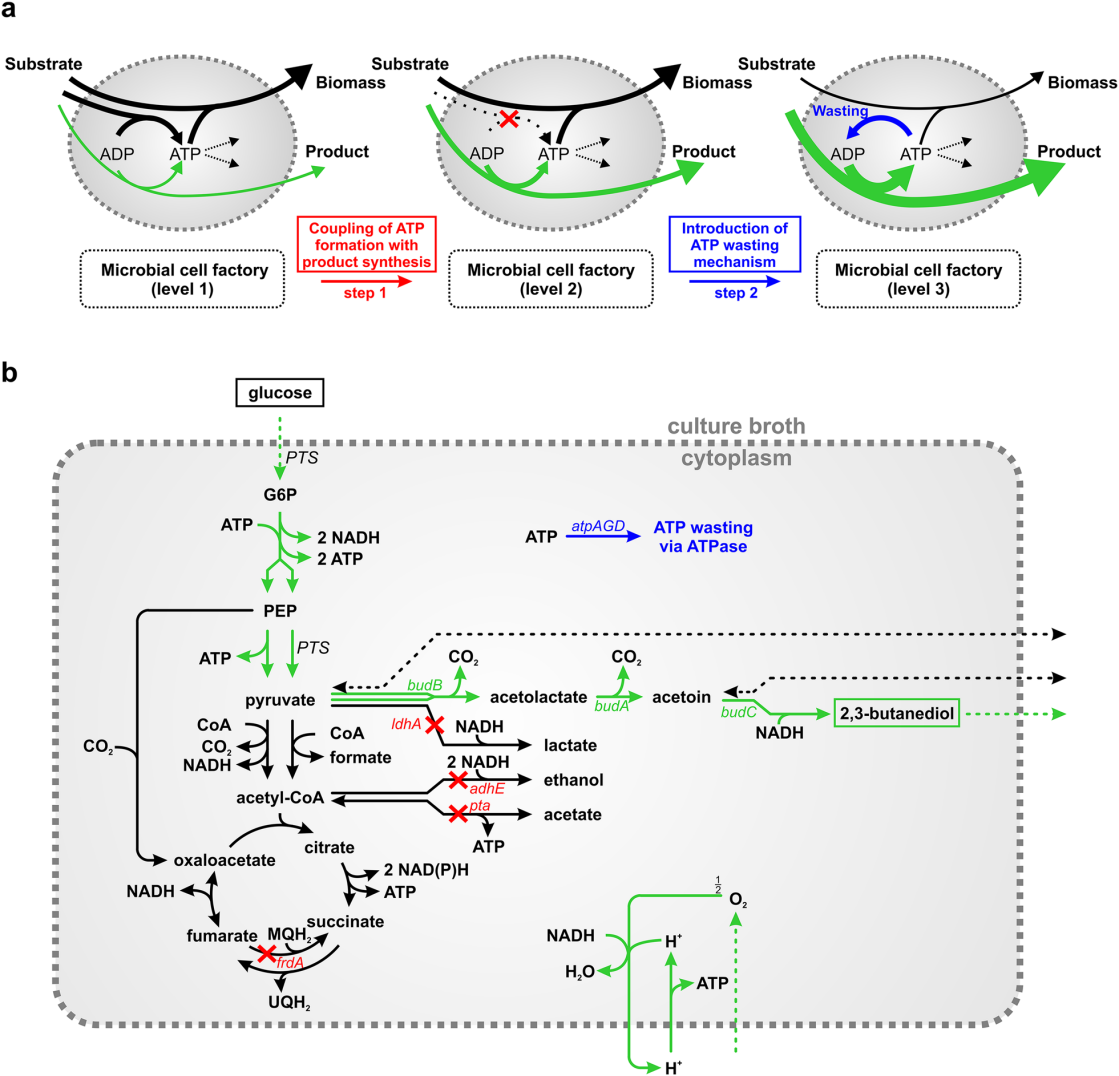
Concept of enforced ATP wasting as metabolic engineering strategy (**a**) and its application to boost 2,3-BDO synthesis in the* E.coli* producer strain 445_Ediss ∆4 (**b**). The green gene names in **b** indicate the heterologous genes that were introduced in [20] to enable 2,3-BDO synthesis. The green reactions indicate a balanced pathway for 2,3-BDO synthesis, which produces ATP and requires oxygen to balance NADH. Red crosses and the corresponding (red) gene names in **b** indicate the deleted pathways to alternative fermentation products in the 445_Ediss ∆4 strain as constructed [20]. With these deletions, ATP synthesis gets coupled with 2,3-BDO synthesis under microaerobic conditions as demanded by step 1 in (**a**). In this study, the F_1_-portion of the ATPase is added as wasting mechanism in this strain representing step 2 in (**a**)

There is an increasing interest in replacing fossil-based synthesis of chemicals with bio-based and sustainable production processes. Several bulk chemicals are already synthesized by cellular factories including, for example, biofuels [1–3], amino acids [4, 5], or organic acids [6]. To be competitive with the petrochemical industry, the three key parameters titer, rate, and yield have to be improved and methods of bioprocess and (systems) metabolic engineering have been developed to build microbial cell factories with desired properties [7]. Most studies in the field focused on improving titer and yield. However, high volumetric productivities are also required to reduce bioreactor size and fermentation time and thus the costs for equipment and process duration [8]. The volumetric productivity depends on biomass concentration, glucose uptake rate, and product yield. Importantly, in growth-coupled production processes, there is an inherent trade-off between biomass formation and product yield, hence, maximal volumetric productivity will need an optimal balance of both. Alternatively, two-stage processes can be used where biomass is produced in the first phase while the product is formed in a growth-decoupled manner in a second stage, ideally with high glucose uptake rate and high product yield [9, 10]. However, a potential problem of two-stage processes is the often low specific activity of growth-arrested cells in the production phase [11, 12]. It has been suggested that enforced ATP wasting could be a suitable strategy to enhance the metabolic activity (i.e., glucose uptake and product formation rate) and thus the volumetric productivity in the production phase [11, 13]. In fact, when enhancing ATP hydrolysis in the cell, e.g. by futile cycles [14, 15] or, as the most direct mechanism, by overexpressing the genes of the uncoupled F_1_-part of the ATPase [16], it has been observed that microorganisms may increase the glycolytic flux and thus the utilization of glucose to compensate for the loss of ATP [16, 13]. If ATP synthesis is coupled to product formation, the increased metabolic activity will necessarily also enhance product synthesis rates. Hence, applying the strategy of enforced ATP wasting requires two steps (Fig. 1a): First, suitable modifications in the metabolic network are identified and implemented (e.g., by knockouts of metabolic genes) with the goal to couple ATP synthesis to the formation of the desired product. In the ideal case, the chemical becomes an obligate byproduct of (stoichiometrically balanced) ATP synthesis. In the second step, an ATP wasting mechanism is introduced in the production host which should boost more flux towards synthesis of ATP and thus of the target product. A positive effect of enforced ATP wasting has been demonstrated for natural fermentation products synthesized by wild type *Escherichia coli* under anaerobic conditions [13] and, more specifically, for the production of acetate [17] and lactate with dedicated *E. coli* strains [15] as well as for synthesis of acetoin with *Lactococcus lactis* [18]. A positive effect of expressing an F_1_-ATPase in *Saccharomyces cerevisiae* could also be shown on ethanol yield, and, under growth-decoupled production, on productivity of ethanol synthesis [19]. However, these previous studies were limited to standard fermentation products of the respective organisms and the improvements in product yield or/and productivity were at most moderate. In this study we go one step further and demonstrate that heterologous production of the alcohol 2,3-butanediol (2,3-BDO) in *E. coli* can be greatly enhanced by enforced ATP wasting.

2,3-BDO is an important compound for the chemical industry. It is used for 1,3-butadiene production which can be further converted to synthetic rubber. Additionally, it finds application in plastics, food, fuel and solvent production [21]. There are three different isomers of 2,3-butanediol: *S,S*; *R,R* and *meso*-2,3-butanediol. Different organisms are natural producers of 2,3-BDO including *Klebsiella pneumoniae* [22], *Klebsiella oxytoca*, and *Paenibacillus polymyxa* [23]. However, these species often require complex medium components and are partially pathogenic hampering their use in industrial applications. Therefore, other organisms have been genetically engineered for 2,3-BDO production, e.g. *E. coli* [20, 24, 25], *Vibrio natriegens* [26], *L. lactis* [27], *S. cerevisiae* [28, 29], *Bacillus subtilis* [30], and *Zymomonas mobilis* [31].

The synthesis of 2,3-BDO starts with the conversion of two molecules of pyruvate to acetolactate by α-acetolactate synthase (Fig. 1b). A following decarboxylation step leads to the formation of acetoin catalyzed by α-acetolactate decarboxylase. Finally, butanediol dehydrogenase reduces acetoin to 2,3-BDO [21] under consumption of one NADH. The heterologous expression (with promotor fine-tuning) of the corresponding genes (*budA*, *budB*, *budC*; Fig. 1b) of these three enzymes of the 2,3-BDO pathway in *E. coli* W allowed the production of 2,3-BDO in glucose minimal-medium (strain 445_Ediss [20]). The authors of [20] then sought to further increase the 2,3-BDO yield of this producer strain. Here it should be noted that, with glucose as substrate, 2,3-BDO production leads to net synthesis of two ATP and one NADH. For a redox-balanced 2,3-BDO synthesis, the NADH generated in excess requires either operation of the electron transport chain with oxygen as electron acceptor (providing further ATP) or alternative fermentation routes as redox sink (Fig. 1b). However, 2,3-BDO synthesis may be disturbed if oxygen is not limited since then all NADH produced in glycolysis might be used to maximize ATP synthesis. Even worse, pyruvate, the precursor of 2,3-BDO, could then also be directed to the TCA-cycle and in combination with the respiratory electron transport chain be converted to CO_2_ and ATP. Therefore, 2,3-BDO synthesis should ideally take place under microaerobic conditions with limited oxygen supply and with blocked pathways to other fermentation products that could act as alternative redox sinks. As a result, ATP synthesis and thus growth become mandatorily coupled to 2,3-BDO synthesis. Accordingly, strain 445_Ediss ∆4 was constructed in [20] where fermentation pathways were blocked by deletion of the *ldhA*, *adhE*, *pta* and *frdA* genes (Fig. 1b). Cultivating this strain under microaerobic conditions with glucose as substrate increased the 2,3-BDO-yield to 0.38 g/g (0.76 mol/mol) in a fed-batch cultivation [20] and the titer could be further increased to 68 g/L. However, the productivity markedly decreased to 1.32 g/(L·h) and therefore requires further optimization.

Since 2,3-BDO synthesis is coupled with formation of ATP in strain 445_Ediss ∆4 (step 1 in Fig. 1a is already accomplished), we postulate that its productivity can be enhanced by imposing enforced ATP wasting (step 2 in Fig. 1a). In the present study, we therefore followed this strategy by overexpressing the genes of the cytosolic F_1_-part of *E. coli*’s ATPase in the strain 445_Ediss ∆4. We tested the performance of this strain under various conditions (aerobic and microaerobic conditions, with and without growth) showing that enforced ATP turnover indeed improves, in all cases, both 2,3-BDO yield and especially specific productivity by a factor of up to 10 compared to a control strain. Based on these results and to avoid microaerobic conditions, we finally propose and validate a three stage-process further increasing the volumetric productivity of 2,3-BDO synthesis by the ATPase strain.

## Results

As described in the Methods, we first transformed the 2,3-BDO producer strain 445_Ediss ∆4 from [20] with a medium copy plasmid (pSB74.5) harbouring the *atpAGD* genes, which encode the F_1_-part of *E. coli*’s F_o_F_1_-ATPase, and put the operon under control of an IPTG-inducible promotor (yielding strain 445_Ediss ∆4_ATPase, in the following called ATPase strain). As has been shown earlier [16], the cytosolic F_1_-portion of the ATPase catalyzes uncoupled ATP hydrolysis and expression of the F_1_-ATPase genes in *E. coli* leads to significant ATPase activity [13]. This mechanism is thus well-suited for enforcing increased ATP turnover in the cell. A control strain (445_Ediss ∆4_control) with the empty vector (pSB76.2) was also constructed for comparison. The performance of both strains under different conditions was tested as described in the following. We used small-scale batch cultivation systems since the main focus of these experiments was to analyze the potential of the ATPase strain to improve productivity of 2,3-BDO synthesis rather than to maximize the product titer.

### Growth‐coupled production under aerobic conditions

**Fig. 2:**
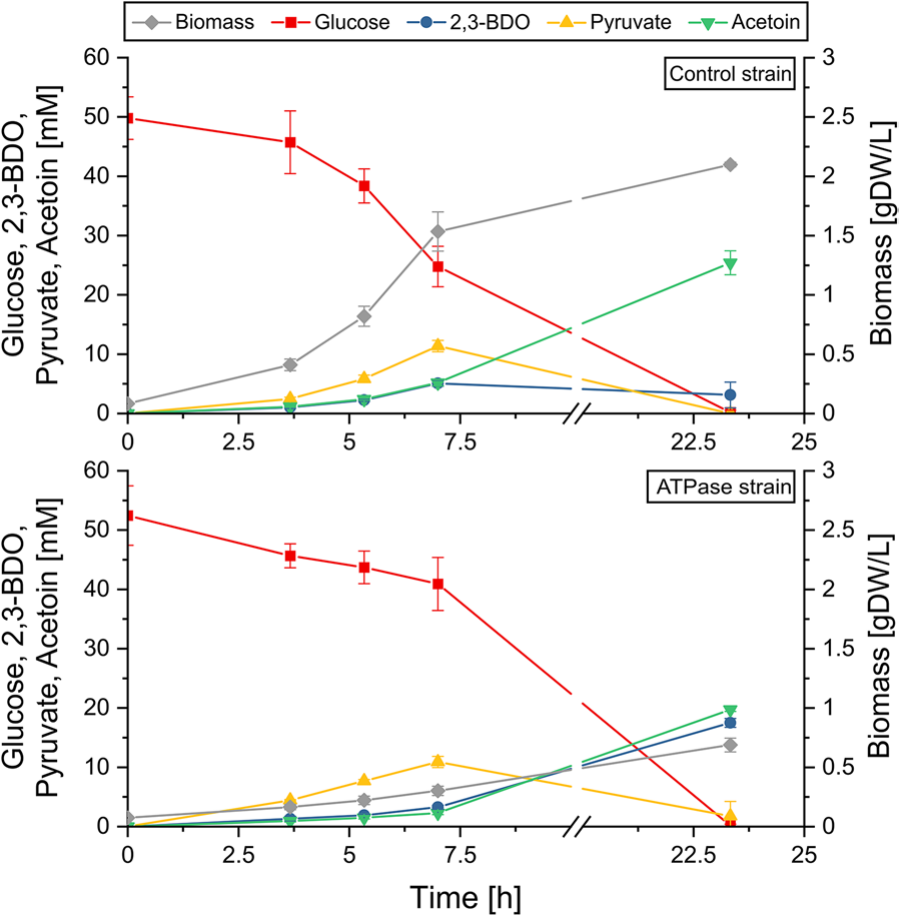
Aerobic cultivation of the ATPase strain and control strain in glucose minimal medium (see also Table 1). The data points are averaged values from triplicates and the error bars indicate standard deviations

In a first setting, both strains were grown under aerobic conditions on minimal medium with 10 g/L glucose and 0.01 mM IPTG. Compared to the control strain, the specific glucose uptake rate of the ATPase strain significantly increased by more than 50 % from 7.52 mmol/(gDW·h) to 11.24 mmol/(gDW·h) (Fig. 2; Table 1). Likewise, the cumulated sum of the product yields of pyruvate, acetoin, and 2,3-BDO increased from 0.87 mol/(mol glucose) to 1.42 mol/(mol glucose). However, the ATPase strain grew with half the growth rate of the control strain and the biomass yield reduced from 0.056 gDW/(mmol glucose) to 0.018 gDW/(mmol glucose). As a consequence, and as expected for a growth-coupled production process, the cultivation with the ATPase strain took longer and its volumetric productivity was 50 % lower than that of the control strain, because the latter has a faster accumulation of the biocatalyst. This disadvantage was addressed by decoupling growth and production as described in the next section.

**Table 1:** Growth rate, glucose uptake rate, specific production rates, product yields and volumetric productivities of the ATPase strain and the control strain in the different cultivation conditions investigated (cf. Figs. 2 and 3 and Additional file 1)

	Exponential growth (aerobic)		Growth arrest by N–limitation (aerobic)		Growth–arrest by N–limitation (microaerobic)	
	Control strain	ATPase strain	Control strain	ATPase strain	ATPase strain	ATPase strain
Time interval for rate and yield calculation [h]	0–7	0–7	0–79	0–7	0–46.5	0–46.5
µ [h^− 1^]	0.42 ± 0.01	0.20 ± 0.0	0*	0	0	0
r_Glucose_ [mmol/(gDW·h)]	7.52 ± 0.88	11.24 ± 0.83	1.28 ± 0.09	9.26 ± 0.51	1.36 ± 0.38	2.20 ± 0.54
r_2,3 − BDO_ [mmol/(gDW·h)]	1.52 ± 0.11	3.18 ± 0.31	0.32 ± 0.02	3.58 ± 1.30	0.96 ± 0.31	1.90 ± 0.46
r_Acetoin_ [mmol/(gDW·h)]	1.55 ± 0.03	2.25 ± 0.17	0.38 ± 0.03	1.55 ± 0.27	0.00 ± 0.00	0.13 ± 0.14
r_Pyruvate_ [mmol/(gDW·h)]	3.34 ± 0.18	10.38 ± 0.28	1.01 ± 0.03	10.22 ± 0.62	0.00 ± 0.00	0.02 ± 0.02
Y_(2,3 − BDO/Glucose)_ [mol/mol]	0.20 ± 0.02	0.29 ± 0.05	0.25 ± 0.03	0.39 ± 0.15	0.70 ± 0.05	0.87 ± 0.05
Y_(Acetoin/Glucose)_ [mol/mol]	0.21 ± 0.03	0.20 ± 0.02	0.30 ± 0.01	0.17 ± 0.03	0.00 ± 0.00	0.05 ± 0.06
Y_(Pyruvate/Glucose)_ [mol/mol]	0.46 ± 0.05	0.93 ± 0.07	0.79 ± 0.05	1.11 ± 0.10	0.00 ± 0.00	0.01 ± 0.01
q_2,3 − BDO_ [mmol/(L·h)]	0.73 ± 0.07	0.47 ± 0.05	0.18 ± 0.03	0.94 ± 0.17	0.45 ± 0.14	0.86 ± 0.20

*Minor growth at the beginning is not considered

The values were calculated from triplicate experiments and the error ranges represent standard deviations. The time intervals for rate and yield calculations were chosen such that they covered the exponential growth phase (growth-coupled production) or the interval where glucose was not yet depleted (under growth arrest)

### Growth‐arrested cells under aerobic and microaerobic conditions

**Fig. 3:**
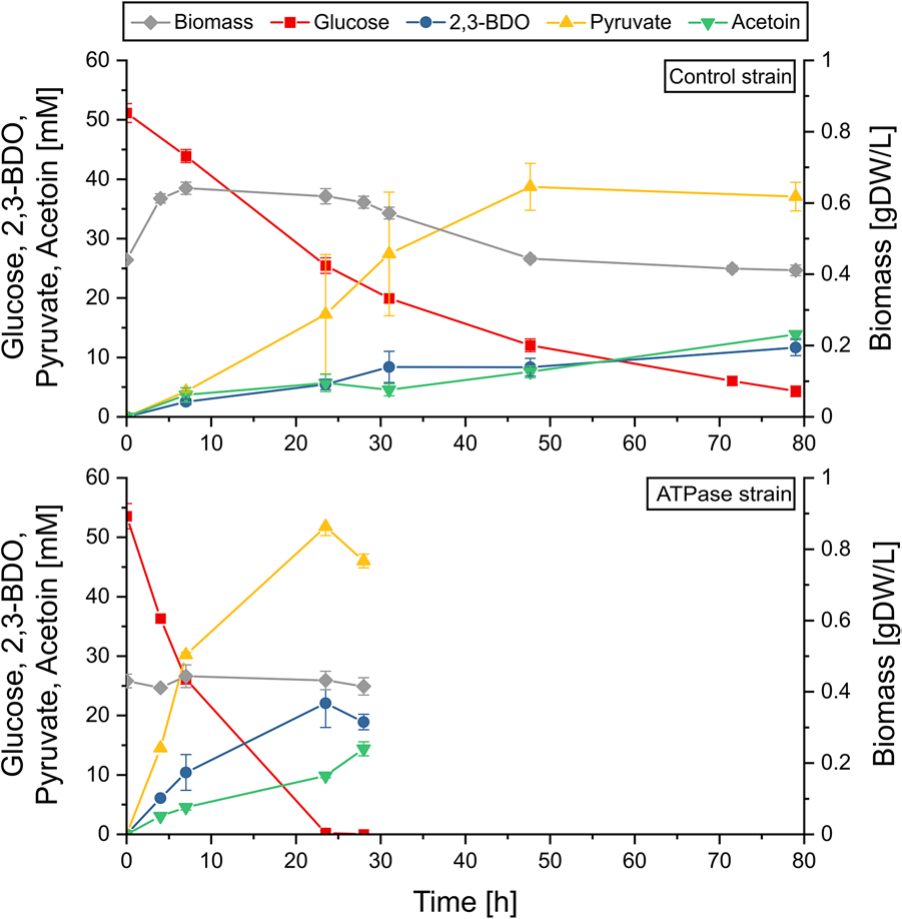
Aerobic cultivation under nitrogen limitation of the ATPase strain and control strain (see also Table 1). Cells were suspended in minimal medium with glucose as substrate and without a nitrogen source. The data points are averaged values from triplicates and the error bars indicate standard deviations

Two-stage processes can be used to resolve the trade-off between growth and volumetric productivity. To analyze the potential of the ATPase strain for this strategy, the two strains were first grown aerobically to accumulate biomass. For the second (production) stage, both strains were growth-arrested and inoculated with an identical OD_420_ of 2 in a medium that lacked a nitrogen source. The control strain showed still limited growth at the beginning of the second phase (Fig. 3), possibly due to some intracellular nitrogen storage or/and due to alteration of the biomass composition [32]. The glucose was rapidly consumed by the ATPase strain with a high uptake rate of 9.26 mmol/(gDW·h), which is close to the uptake rate under growth and six times higher than the rate of the control strain under these conditions. Therefore, despite the higher biomass concentration of the control strain, glucose was depleted much faster and the volumetric productivity of 2,3-BDO increased fourfold and the specific 2,3-BDO synthesis even tenfold in the ATPase strain. When glucose is exhausted, the cells start to generate ATP by reutilizing the produced 2,3-BDO and oxidizing it to acetoin yielding NADH, which can fuel electrons to the respiratory chain (Fig. 3). Hence, the process should be stopped at this time point to avoid consumption of the product 2,3-BDO.

Compared to growth-coupled production, the combined yield of pyruvate, acetoin and 2,3-BDO by the ATPase strain also increased by 25 % to 1.67 mol/(mol glucose). However, the (undesired) high yield of pyruvate (1.11 mol/(mol glucose)) shows that the conversion of pyruvate to 2,3-BDO via acetoin is used only to a lower extent by the cells. Indeed, although 2,3-BDO synthesis leads to net production of ATP needed by the cells to compensate for the enforced ATP dissipation, the continued conversion of pyruvate to 2,3-BDO consumes NADH. This reduces the ATP yield because NADH can alternatively be oxidized via the respiratory chain delivering further ATP as discussed earlier (Fig. 1b).

Therefore, as the next step, we performed again a growth-arrested cultivation but this time under microaerobic conditions, where NADH excess should increase 2,3-BDO synthesis to balance redox equivalents (see Additional file 1). As intended, both strains now produced mainly 2,3-BDO with a high yield of 0.87 mol/(mol glucose) (or 0.44 g/(g glucose)) for the ATPase strain and a reduced yield of 0.70 mol/(mol glucose) for the control strain (Additional file 1 and Table 1). The specific glucose uptake rate was 62 % higher with the ATPase strain compared to the control strain. However, compared to the aerobic conditions, the glucose uptake was significantly reduced in both strains leading to reduced volumetric productivities. Apparently, oxygen became now a limiting factor: for each molecule of 2,3-BDO produced, only one of the two NADH generated in glycolysis are consumed and therefore 0.5 molecules of O_2_ are needed by the cell to remove the second NADH via respiration (see Fig. 1b). Furthermore, although the trends were consistent in all replicates, we also observed a relatively high standard deviation in the measured substrate and product concentrations indicating that the tight range of microaerobic oxygen concentrations is difficult to maintain in our chosen setup. While fed-batch cultivations in bioreactors as used in [20] may allow a better control of the DO, microaerobic process conditions should generally be avoided for industrial applications, as a stable and homogeneous adjustment of low oxygen levels is usually very difficult in large-scale bioreactors.

### Three‐stage cultivation for optimal 2,3-BDO synthesis

We therefore conceived a three-stage process, which does not require microaerobic conditions but still harnesses the increased metabolic activity of the ATPase strain for synthesizing 2,3-BDO with both high productivity and high yield. In stage 1, biomass is produced in sufficient amounts under aerobic conditions. With the inducible promotor, expression of the F_1_-ATPase could be blocked in the ATPase strain in stage 1 to ensure high biomass yields while it will be induced in the two subsequent stages. Stage 2 and 3 are production phases (without growth), first under aerobic (stage 2) and then under anaerobic (stage 3) conditions. In the second stage, 2,3-BDO and the main by-products acetoin and pyruvate will be produced in a growth-decoupled manner (nitrogen limitation) with oxygen as potential electron acceptor. According to the result from previous experiments in Table 1, the use of the ATPase strain should already increase the product yield and even more the productivity (the latter by a factor of 5–10) in this second phase. The aim of the third and last stage is to also convert the by-products pyruvate and acetoin (which are both intermediates in the 2,3-BDO pathway; Fig. 1b) to 2,3-BDO. As discussed above, anaerobic conversion of glucose to 2,3-BDO is not feasible due to redox imbalance (one NADH is produced in excess). However, simultaneous uptake of acetoin and/or pyruvate and their conversion to 2,3-BDO may serve as the required electron sink. With acetoin as co-substrate, the net conversion would be

$$glucose\;+\;acetoin\;+\;2\;ADP\;+\;2\;Pi\rightarrow2\;(\text{2,3-BDO})\;+\;2\;ATP\;+\;2\;CO_2$$

and with pyruvate it reads

$$glucose\;+\;2\;pyruvate\;+\;2\;ADP\;+\;2\;Pi\rightarrow2\;(\text{2,3-BDO})\;+\;2\;ATP\;+\;4\;CO_2.\;$$

With known product yields for the aerobic growth-decoupled production phase (Table 1) and with a given start concentration of glucose, one can calculate at which glucose concentration one needs to switch from the aerobic to the anaerobic production phase, such that the remaining glucose matches the amount stoichiometrically needed to co-consume the by-products acetoin and pyruvate excreted in the aerobic phase and to finally convert them to 2,3-BDO.

**Fig. 4:**
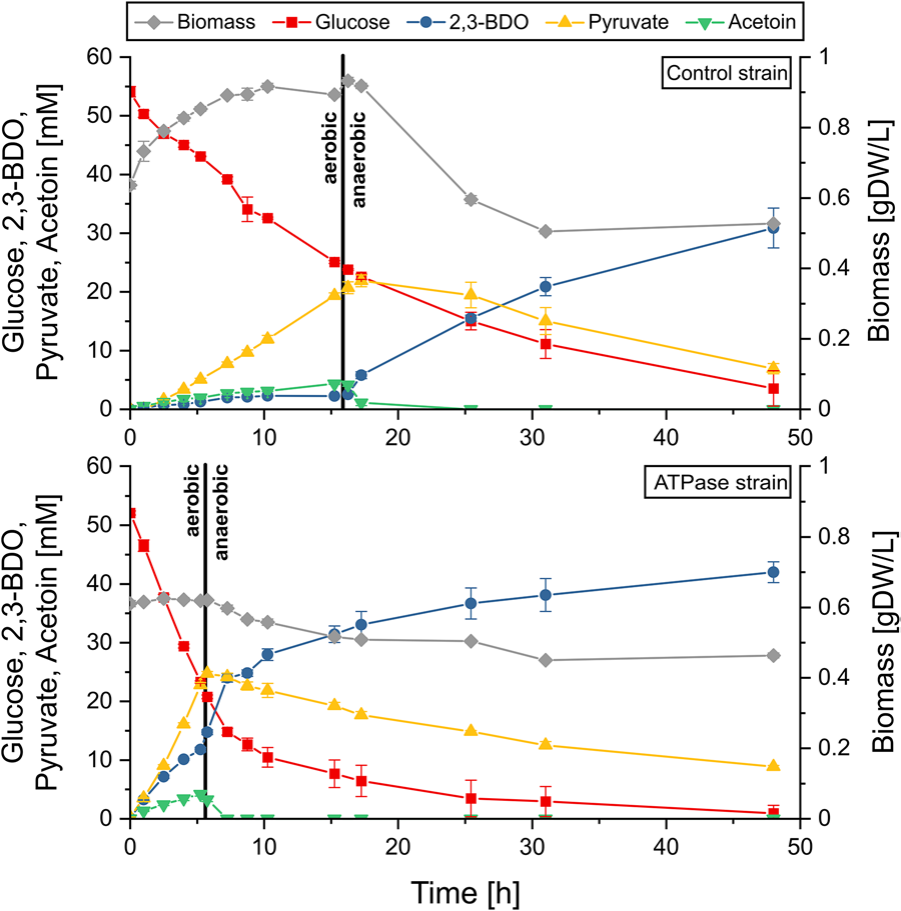
Results for the second (aerobic, no growth) and third (anaerobic, no growth) phase of a three-stage process for 2,3-BDO production with the ATPase strain and the control strain (see also Table 2). When switching from aerobic to anaerobic conditions the culture were transferred from shake flasks (250 rpm) to glass bottles incubated in an anaerobic chamber. The data points are averaged values from triplicates and the error bars indicate standard deviations

We performed this three-stage process with both the ATPase and the control strain (see Methods). As a minor simplification, the precultures grown under aerobic conditions were considered as the first (aerobic growth) stage, so that the actual process shown in Fig. 4 started—with sufficient amounts of biomass (OD_420_ ≈3 for both strains)—at the second stage, i.e. with the aerobic production under growth arrest. With a glucose start concentration of 10 g/L (55 mmol/L) and the estimated product yields for this regime from Table 1, we calculated that the optimal switch point is reached when 58 % (ATPase strain) or 59 % (control strain) of the glucose has been consumed. We accordingly switched from aerobic to fully anaerobic conditions when the respective amount of glucose was consumed by the strains (vertical lines in Fig. 4).

**Table 2:** Yields and rates of the three-stage process for 2,3-BDO production with the ATPase strain and the control strain (see also Fig. 4)

	Stage 2: Aerobic phase (growth arrest by N limitation)		Stage 3: Anaerobic phase (growth arrest by N limitation)				Overall process (aerobic and anaerobic phase under growth arrest)	
			With acetoin consumption		Acetoin depleted			
	Control strain	ATPase strain	Control strain	ATPase strain	Control strain	ATPase strain	Control strain	ATPase strain
Time interval [h]	0–15.25	0–5.25	16.25–17.25	5.75–7.25	25.42–48	7.25–25.42	0–48	0–25.42
r_Glucose_ [mmol/(gDW·h)]	2.30 ± 0.09	8.86 ± 0.19	1.33 ± 0.34	6.51 ± 0.93	0.94 ± 0.12	1.15 ± 0.25	1.35 ± 0.07	3.30 ± 0.17
r_2,3-BDO_ [mmol/(gDW·h)]	0.18 ± 0.00	3.60 ± 0.08	3.54 ± 0.69	10.14 ± 1.08	1.25 ± 0.25	1.28 ± 0.22	0.83 ± 0.09	2.48 ± 0.17
r_Acetoin_ [mmol/(gDW·h)]	0.35 ± 0.01	1.28 ± 0.09	− 3.29 ± 0.56	− 3.58 ± 0.34	0.00 ± 0.00	0.00 ± 0.00	0.00 ± 0.00	0.00 ± 0.00
r_Pyruvate_ [mmol/(gDW·h)]	1.54 ± 0.04	6.93 ± 0.09	1.29 ± 0.38	− 0.63 ± 0.53	− 1.02 ± 0.10	− 0.95 ± 0.05	0.19 ± 0.02	0.99 ± 0.00
Y_(2,3-BDO/Glucose)_ [mol/mol]	0.08 ± 0.00	0.41 ± 0.02	2.73 ± 0.49	1.57 ± 0.08	1.32 ± 0.10	1.13 ± 0.06	0.61 ± 0.04	0.75 ± 0.01
Y_(Acetoin/Glucose)_ [mol/mol]	0.15 ± 0.01	0.14 ± 0.01	− 2.63 ± 0.71	− 0.56 ± 0.06	0.00 ± 0.00	0.00 ± 0.00	0.00 ± 0.00	0.00 ± 0.00
Y_(Pyruvate/Glucose)_ [mol/mol]	0.67 ± 0.02	0.78 ± 0.02	1.10 ± 0.60	− 0.11 ± 0.09	− 1.10 ± 0.03	− 0.86 ± 0.16	0.14 ± 0.01	0.30 ± 0.01
q_2,3-BDO_ [mmol/(L·h)]	0.15 ± 0.00	2.23 ± 0.06	3.27 ± 0.64	6.18 ± 0.68	0.68 ± 0.15	0.70 ± 0.13	0.64 ± 0.07	1.44 ± 0.10

Shown are the results for the (growth-decoupled) production stages 2 and 3. The switch from aerobic to anaerobic conditions was initiated when approximately 60% of the glucose had been consumed (see text). Negative rates and yields for acetoin and pyruvate indicate uptake of the respective compound. The values were calculated from triplicate experiments and the error ranges represent standard deviations.

The results of this experiment validate the proposed approach (Fig. 4; Table 2) and emphasize again the boosting effect of increased ATP turnover. A significant difference in the productivity of both strains can be observed. As in the single-stage cultivation, the ATPase strain exhibits a very high metabolic activity under aerobic conditions, despite the growth arrest due to nitrogen limitation. Already after 5.3 hours, nearly 60 % of the glucose was consumed and the switch to anaerobic production could be performed. As expected, in the third phase, acetoin and pyruvate were then taken up together with glucose, mainly to synthesize ATP consumed by the F_1_-ATPase and other (non-growth associated) maintenance processes. Acetoin is taken up very quickly (it is depleted within 3 hours after the switch to anaerobic conditions) and converted to 2,3-BDO leading to the highest productivity of 2,3-BDO synthesis during the entire process. Afterwards, pyruvate is taken up as co-substrate with glucose, but with a reduced rate compared to acetoin. Generally, the co-consumption of acetoin and pyruvate in the anaerobic phase increases the 2,3-BDO/glucose yield beyond the maximum value of 1 mol/mol normally achievable with glucose under aerobic conditions. After 25.4 h, more than 93 % of the glucose had been consumed by the ATPase strain with an overall 2,3-BDO-yield of 0.75 mol/(mol glucose) (75 % of the maximum yield) and with relatively little pyruvate remaining in the medium. For the sake of a high overall volumetric productivity, in a realistic process one would probably stop the process at this point or even earlier (after 17 h). Alternatively, the process could be continued for a longer period to fully deplete substrate and pyruvate. The molar ratio of pyruvate and glucose in the medium after acetoin depletion is slightly above the optimal value of 2. Moreover, the actual uptake ratio is close to 1 and thus below the expected value of 2 at which redox balance can be achieved (one possible explanation could be that other oxidized components from the medium serve as redox sinks, e.g. originating from cell lysates (see biomass reduction in Fig. 4)). For these two reasons, some pyruvate remains in the medium. To maximize 2,3-BDO yield, the optimal switching point should therefore be chosen slightly earlier for full depletion of both glucose and pyruvate.

In comparison, the control strain shows a similar overall behavior, but with much smaller specific and volumetric rates, especially in the aerobic phase. For example, despite the fact that the strain grew slightly further in the nitrogen-free medium until 10 hours, the specific and volumetric substrate uptake and product synthesis rate were markedly lower than in the ATPase strain (Table 2; also product yields were smaller). Therefore, the process with the control strain could be switched from aerobic to anaerobic production only after 16.25 hours, more than 10 hours later than the ATPase strain. As in the ATPase strain, acetoin is quickly co-consumed with glucose in the beginning of the anaerobic phase resulting in high 2,3-BDO yield. Here, the maximum yield of 2 mol 2,3-BDO per mol glucose achievable under co-consumption of acetoin or/and pyruvate is even exceeded in this short phase, which might indicate that the cell utilizes additional redox equivalents (e.g., generated from storage compounds from the previous phase). Again, the first short (acetoin consumption) sub-phase of stage 3 is followed by a longer second sub-phase with a reduced glucose and pyruvate uptake rate. Only after 48 hours, the control strain reaches a remaining glucose level that is comparable with the glucose concentration reached by the ATPase strain after 25 hours. The final overall 2,3-BDO yield and titer are also lower compared to the ATPase strain. This can be attributed to the reduced 2,3-BDO, acetoin and pyruvate yield in the aerobic phase, where the percentage of the glycolytic flux entering the TCA cycle (with subsequent use of the respiratory chain) is most likely higher than in the ATPase strain. In the latter, due to the increased ATP demand, overflow metabolism via the 2,3-BDO pathway and via pyruvate excretion seems to become more important relative to the TCA cycle, which is another advantage of the ATPase strain.

## Discussion

In this work we employed enforced ATP wasting as a strategy to increase the productivity of 2,3-BDO synthesis in a dedicated *E. coli* producer strain. At a first glance, it might appear unintuitive why wasting of ATP—the main energy currency required for numerous processes in the cell—can help to design efficient cell factories. But if the metabolic pathway to a desired product contributes significantly to net ATP synthesis in the cell, an increased drain of ATP may be beneficial for two reasons: it may (i) thermodynamically favor a higher flux along this pathway and (ii) trigger a (desired) upregulation of the flux along the ATP-synthesizing pathway by the microorganism itself. Since the pathway from glucose to 2,3-BDO generates ATP via glycolysis, we postulated that a higher drain of ATP could further boost heterologous production of 2,3-BDO synthesis in the *E. coli* producer strain.

The results of the present study confirmed this prediction and markedly positive effects of enforced ATP turnover on 2,3-BDO synthesis could be observed under all tested cultivation conditions. We constructed a strain that expresses the genes of the cytosolic and ATP-hydrolyzing F_1_-ATPase from a plasmid in the 2,3-BDO producer strain 445_Ediss ∆4. As a first proof of principle, during exponential growth under aerobic conditions, this ATPase strain indeed increased its specific glucose uptake and 2,3-BDO synthesis rates by 50 % and the 2,3-BDO yield by 45%. However, the decreased growth rate and biomass yield reduced the amount of available biocatalyst and thus the volumetric productivity. This disadvantage of growth-coupled production can be overcome by two-stage processes and we therefore used nitrogen limitation to stop aerobic growth and to investigate the behavior of the strains in a pure production phase. Specific glucose uptake rates under growth arrest are typically below 1 mmol/(gDW·h) (see e.g. [33]) hampering the application of two-stage processes and a similarly low glucose uptake rate of 1.28 mmol/(gDW·h) was indeed observed in our control strain. Previous works could enhance the specific glucose uptake rate in *E. coli* under nitrogen starvation to 2.5 mmol/(gDW·h) by overexpression of parts of the glucose transport system [34] and to 3.33 mmol/(gDW·h) by modulating the stringent response [35]. Using enforced ATP wasting we could recently further increase the glucose uptake rate in resting *E.coli* cells to 6.78 mmol/(gDW·h) [13]. The rate of 9.26 mmol/(gDW·h) observed for our 2,3-BDO-strain with ATPase was even 35 % higher and is, to the best of our knowledge, the highest value ever reported for non-growing *E. coli* cells. Compared to the control strain, this was a more than sixfold increase. Likewise, the specific and volumetric product synthesis rates were elevated by a factor of 10 and 4, respectively. However, the 2,3-BDO yield was still rather low and the high amount of pyruvate produced indicated that the pathway from pyruvate to 2,3-BDO was inefficient under aerobic conditions, presumably because a larger fraction of NADH was consumed via the respiratory chain instead for reduction of acetoin. This is a general disadvantage of using aerobic conditions for 2,3-BDO synthesis, even in conjunction with enforced ATP wasting: while production of 2,3-BDO is coupled to net ATP synthesis, the other direction holds not true, since ATP synthesis is also possible without 2,3-BDO formation.

For applications of enforced ATP wasting, obligate coupling, where balanced ATP synthesis is only feasible with product formation, is the ideal case. Computational strain design methods have been developed to identify intervention strategies that lead to such obligate coupling of ATP (or, alternatively, biomass) synthesis with product formation [36, 37]. One possible way to achieve strong coupling of ATP and 2,3-BDO synthesis is to use microaerobic instead of fully aerobic conditions where, on the one hand, the NADH supply for 2,3-BDO synthesis increases due to limited oxygen availability, while the amount of supplied oxygen exactly balances the remaining surplus of NADH when producing 2,3-BDO from glucose. For this reason, microaerobic cultivations have frequently been used for 2,3-BDO synthesis [20, 24, 29, 30] and we therefore tested the effect of ATP wasting also for this regime. First of all, applying microaerobic conditions under growth arrest indeed doubled the 2,3-BDO yield in the ATPase strain to 87 % of the maximum yield, which is the highest yield ever reported for *E. coli* on minimal glucose medium. Compared to the control strain under these conditions, both yield (+ 24 %) as well as glucose uptake rate (+ 60 %) were again significantly increased in the ATPase strain. However, the glucose uptake rate and, therefore, despite the high 2,3-BDO yield, also the specific and volumetric productivity of the ATPase strain (and likewise of the control strain) dropped markedly compared to fully aerobic conditions. Further investigations are required to understand why the specific rates (both substrate uptake and 2,3-BDO synthesis) in the microaerobic cultivation do not reach that of the aerobic counterpart.

To circumvent these adverse effects and since microaerobic conditions are also difficult to adjust and control in large-scale bioreactor applications, we finally proposed and tested a completely new cultivation strategy for 2,3-BDO synthesis. This strategy is based on a three-stage process, which again benefits from enforced ATP wasting and enables higher productivity also in combination with relatively high product yield. After an aerobic growth phase, the second aerobic phase under growth arrest is devoted to synthesizing large amounts of 2,3-BDO and, due to incomplete substrate conversion, of its precursors pyruvate and acetoin. As was already seen in the (single-stage) aerobic cultivation under nitrogen starvation (Table 1), ATP wasting enormously boosts the efficiency of this second phase (Table 2), especially in terms of specific and volumetric rates but also in terms of yields. In the third phase, the precursor products acetoin and pyruvate are co-consumed with glucose to yield more 2,3-BDO. The anaerobic conditions in the third stage (together with the knockouts of the alternative fermentation routes) ensure that 2,3-BDO and ATP synthesis are now mutually fully coupled. A remarkable difference in the co-consumption rates of acetoin and pyruvate could be observed, which virtually divide the third phase into two sub-phases (Table 2). Acetoin is very quickly consumed with glucose leading to the highest specific 2,3-BDO synthesis rates of all cultivations and stages. Co-consumption of pyruvate and glucose is much slower indicating that the reactions of α-acetolactate synthase and/or α-acetolactate decarboxylase could be limiting steps. This might be overcome by further optimizing the expression ratios of these enzymes in the 2,3-BDO pathway (or by using alternative α-acetolactate synthases, see e.g. [38]) to increase the conversion of pyruvate to 2,3-BDO or at least to acetoin in the second (aerobic) stage. ATP wasting also increases substrate uptake and 2,3-BDO synthesis rates in the third phase but has favorable effects mainly during the first sub-phase with acetoin consumption.

An interesting extension of the proposed three-stage process would be a fed-batch operation cycling between the second (aerobic) and third (anaerobic) phase with glucose feeding at the beginning of each aerobic phase. This could further increase the 2,3-BDO titer, which was not the main objective of this paper, since herein we focused on the relative performance gain of the ATPase strain in a small-scale cultivation system with minimal glucose medium. Likewise, the obtained volumetric productivities of the two-stage or three-stage cultivations could easily be enhanced by starting with a higher biomass concentration at the beginning of the production phase(s), ideally in bioreactors. In this regard, it is noteworthy that the specific productivity of the ATPase strain in the presented three-stage process is 50 % higher than that of the high-performance producer strain 445_Ediss ∆4 used in the microaerobic fed-batch process in [20]. Thus, since the specific productivity governs the volumetric rate of growth-decoupled production phases, superior volumetric productivities can be expected for the ATPase strain in the three-stage process if comparable biomasses concentrations are used. Finally, the proposed three-stage process as well as the strategy of enforced ATP wasting in general could also be used to further enhance other developed production hosts and/or processes for 2,3-BDO synthesis published in the literature [24, 29, 30].

## Conclusions

We could show that enforced ATP wasting is a suitable tool to significantly improve 2,3-BDO synthesis in *E. coli.* Although the ATPase strain showed increased 2,3-BDO yields under all tested conditions, we believe that, compared to previous achievements, the highest potential of enforced ATP wasting is to maximize the volumetric productivity of non-growing cells in processes where growth and production are separated. Our study achieved the highest productivity improvements so far reported in combination with enforced ATP wasting and we expect more applications of this generic metabolic engineering strategy to come in the future. Apart from additional case studies, important aspects for future research concern the optimal (expression) level of the ATPase and thus of the optimal amount of drained ATP. In this study we used a plasmid with medium copy number and an inducer (IPTG) concentration, which together turned out to be suitable to demonstrate beneficial effects of ATP wasting for 2,3-BDO synthesis, but we did not further optimize it. We expect that, specific for the used host organism, product and process, there will be an optimum beyond which adverse effects will reduce the performance of the strain. This could become a nice showcase for dynamic metabolic control strategies [39] to fine-tune the ATPase expression level at which maximal product yield or/and productivity can be achieved. This could also be combined with metabolic switches based on quorum sensing [40, 41] to avoid the use of expensive external inducers when moving from growth to production phase.

## Materials and methods

### Strains and plasmid construction

All strains, plasmids and primers used in this study are summarized in Additional file 1: Table S1. All cloning steps and plasmid propagation were performed in *E. coli* NEB 5-alpha cells (New England Biolabs). The F_1_-ATPase encoding genes *atpAGD* were amplified from plasmid pCP41::*atpAGD* [16] by PCR using the Q5 Hot Start High Fidelity Polymerase (New England Biolabs) and primer pair atpAGD_mono_fw/atpAGD_mono_rv according to the manufacturer’s instructions. The amplicon as well as plasmid pSB-T2g [42] were digested with the restriction enzymes NdeI (New England Biolabs) and BamHI-HF (New England Biolabs). The *atpAGD*-amplicon was ligated into the backbone of pSB-T2g using the T4 DNA ligase (New England Biolabs). The pMB1 replicon was then cut out from the constructed plasmid (using restriction enzymes SpeI-HF (New England Biolabs) and AscI (New England Biolabs)) and replaced by the p15A replicon which was amplified from plasmid pZA31-luc [43] by PCR using the Q5 Hot Start High Fidelity Polymerase (New England Biolabs) and primer pair p15A_SpeI_fw/p15A_AscI_rv. To be compatible with the 2,3-BDO-pathway harboring plasmid BB3_pUC(Kan^R^)_445_Ediss, the kanamycin resistance cassette from the constructed F_1_-ATPase plasmid was replaced by an ampicillin resistance cassette: The fragment of the ATPase plasmid lacking the kanamycin resistance cassette was amplified by PCR using primer pair pSB_MT_backbone_fw/pSB_MT_backbone_rv. The ampicillin resistance cassette was amplified by PCR from plasmid pSB38.2 [13] using primer pair Amp_Gibson_fw/Amp_Gibson_rv. Both PCR fragments were assembled using Gibson assembly [44] to yield plasmid pSB74.5. To construct the control plasmid pSB76.2, the F_1_-ATPase encoding genes *atpAGD* were cut out from plasmid pSB74.5 using restriction enzymes NdeI (New England Biolabs) and BamHI-HF (New England Biolabs). The 5’-overhangs were filled-in using the Klenow fragment (Thermo Scientific) and the blunt-ended fragment was self-ligated.

The strain 445_Ediss ∆4 was transformed with the plasmids pSB74.5 and pSB76.2 by CaCl_2_ (0.1 M) and heat shock (90 sec 42 °C) to yield strains 445_Ediss ∆4 ATPase and 445_Ediss ∆4 control, respectively.

### Media and cultivation

Strains were first grown in LB_0_-medium (10 g/L tryptone, 5 g/L yeast extract and 5 g/L NaCl) for 5–7 h at 37 °C. A second preculture consisting of minimal medium (MM, adapted from [45]) supplemented with 4 g/L glucose and antibiotics (kanamycin 50 µg/L and ampicillin 100 µg/L) and 0.01 mm IPTG was inoculated 1:100 from the LB_0_-culture and cultivated at 37 °C and 250 rpm in shake flasks (three baffles, 10 % fill volume) over night. To inoculate all main cultures (shown in Figs. 2, 3 and 4 and Additional file 1: Fig. S1, cells from the MM-preculture were centrifuged (5000 x* g*, 10 minutes, 20 °C) and resuspended to the same OD for the ATPase and control strain in fresh MM (supplemented with 50 µg/L kanamycin, 100 µg/L ampicillin and 0.01 mm IPTG) with 10 g/L glucose as substrate. For aerobic cultivation, 250 mL-shake flasks with three baffles and a filling volume of 10 % were shaken at 250 rpm at 37 °C. For microaerobic cultivation, the flasks were incubated at 37 °C without shaking. Anaerobic cultivation was performed in 50 ml glass bottles (filled with 25 mL of medium), which were placed into an anaerobic work chamber (Don Whitley Scientific) with an oxygen-free atmosphere (80 % N_2_, 10% CO_2_, 10% H_2_) and stirred at 37 °C. For cultivation without cell growth, MM without nitrogen source was used, where (NH_4_)_2_SO_4_ was replaced by Na_2_SO_4_.

The three-stage cultivation process was performed as follows: 50 mL of MM (supplemented with 50 µg/L kanamycin, 100 µg/L ampicillin and 0.01 mm IPTG) with 10 g/L of glucose were inoculated from the LB_0_-preculture as described above and cultivated in 500 mL-shake flasks (three baffles) at 37 °C and 250 rpm overnight (stage 1, not shown in Fig. 4). Cells were centrifuged (5000 x g, 10 minutes, 20 °C), washed and resuspended to an OD_420_ of 3.0 into 25 ml of fresh MM (without nitrogen source, supplemented with 50 µg/L kanamycin, 100 µg/L ampicillin and 0.01 mm IPTG) and 10 g/L glucose and cultivated aerobically in 250 mL-shake flasks as described above (stage 2). At the indicated time points, cultures were then transferred into 50 ml glass bottles and cultivated anaerobically as described above (stage 3).

All cultivations were performed in triplicates.

### Analytics, enzyme assays

Glucose concentrations were measured by the HK assay kit (Megazyme Ltd.). Pyruvate, acetoin and 2,3-BDO were quantified by HPLC using an UV and RI-detector. The column Rezex ROA-Organic Acid H+ (8 %) (Phenomenex) was operated at 65 °C with a flow rate of 0.5 mL/min and a running buffer consisting of 4 mm H_2_SO_4_.

### Yield and rate calculations

Biomass concentration was monitored by measuring the optical density at 420 nm (OD_420_) and using a conversion factor of 0.22 to calculate the biomass concentration in gDW/L. Specific uptake and excretion rates for the exponential phase in growth-coupled experiments were determined with the formula.

$$r{\mkern 1mu} = \mu {\mkern 1mu} (c_{M,e} - c_{M,s}){\mkern 1mu} /{\mkern 1mu} (c_{X,e} - c_{X,s}){\mkern 1mu} [mmol/(gDW \cdot h)]$$

where *µ* is the growth rate, *c*_*M,e*_ and *c*_*M,s*_ represent the end and start concentrations of the respective metabolite M (mmol/L glucose, 2,3-BDO, acetoin, or pyruvate) and *c*_*X,e*_ and *c*_*X,s*_ represent the end and *s*tart concentrations of the biomass (gDW/L). In experiments with growth arrest, where the biomass concentration remained nearly constant, the specific rates are calculated as.

$$r\,=\,(c_{M,e}-c_{M,s})/(X_{Av}\cdot\Delta t)\lbrack mmol/(gDW\cdot h)\rbrack$$

where *X*_Av_ is the average biomass concentration (gDW/L), and Δ*t = t*_*e*_ – *t*_*s*_ the length of the time period (difference of end and start time). For the average volumetric productivity of a metabolite M (in both growth-coupled and growth-decoupled cultivations) we used the formula.

$$q\,=\,(c_{M,e}-c_{M,s})/\Delta t\,\lbrack mmol/(L\cdot h)\rbrack.$$

Overall yields were calculated by taking the first and last time point of the respective production phase into account. The respective time period used for the calculations of rates, productivities, and yields are mentioned in the text or/and tables.

## Supplementary Information

**Additional file 1.** Results of microaerobic cultivations; Strains, plasmids and primers used in this study. 

## Data Availability

The authors declare that all data supporting the findings of this study are available within the paper and its Supplementary Information files or are available from the corresponding author on request.
