# Supplementary material for: Increasing ATP turnover boosts productivity of 2,3-butanediol synthesis in Escherichia coli
Source: Microb Cell Fact. 2021 Mar 9;20:63. doi: 10.1186/s12934-021-01554-x (PMC7941745; doi:10.1186/s12934-021-01554-x)
Supplement: Supplementary file 1 — Additional file 1. Results of microaerobic cultivations; Strains, plasmids and primers used in this study. [file 12934_2021_1554_MOESM1_ESM.docx]

Additional File 1

**Increasing ATP Turnover Boosts Productivity of
2,3-Butanediol Synthesis in *Escherichia coli***

Simon Boecker^1,#^, Björn-Johannes Harder^1,#^, Regina Kutscha^2^,
Stefan Pflügl^2^, Steffen Klamt^1,*^

^1^Max Planck Institute for Dynamics of Complex Technical Systems, Sandtorstraße 1, 39106 Magdeburg, Germany

^2^Technische Universität Wien, Institute for Chemical, Environmental and Bioscience Engineering, Research Area Biochemical Engineering, Gumpendorfer Straße 1a, 1060 Vienna, Austria

^*^Corresponding author: [klamt@mpi-magdeburg.mpg.de](mailto:klamt@mpi-magdeburg.mpg.de)

^#^These authors contributed equally **Supplementary Text 1:** Microaerobic cultivation

For inducing microaerobic conditions, the cultures were not shaken, which strongly reduced the dissolved oxygen in the medium below 0.1% (similar to the microaerobic bioreactor cultivations in [1]. For further discussion see main text and Table 1.


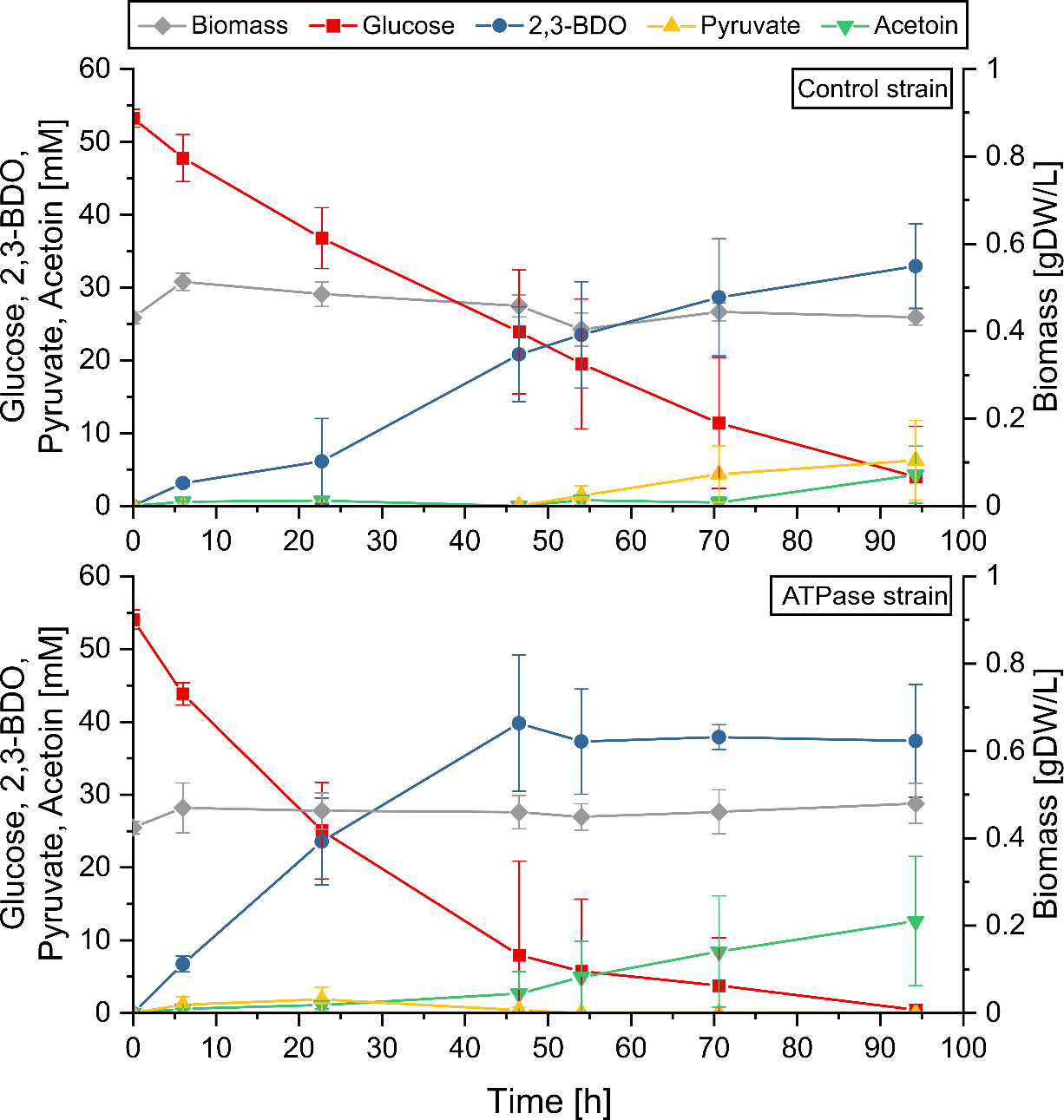


**Supplementary Figure 1**: **Microaerobic cultivation under nitrogen limitation of the ATPase strain and the control strain (see also Table 1 in main text)**. Minimal medium with glucose as substrate and without nitrogen source. Cultivation without shaking. The data points are averaged values from triplicates and the error bars indicate standard deviations. For further discussion see main text and Table 1.

**Supplementary Table 1**: Strains, plasmids and primers used in this study

| **Strain or plasmid** | **Relevant characteristics** | **Source** |
| --- | --- | --- |
| *E. coli* NEB 5-alpha | Competent cells for cloning and plasmid propagation | New England Biolabs, #C2987U |
| 445_Ediss ∆4 | *E. coli* W ∆*ldhA* ∆ *adhE* ∆*pta* ∆*frdA* transformed with construct BB3_pUC(Kan^R^)_445_Ediss | [1] |
| 445_Ediss ∆4_ATPase | 445_Ediss ∆4 transformed with pSB74.5 | This study |
| 445_Ediss ∆4_control | 445_Ediss ∆4 transformed with pSB76.2 | This study |
| pCP41::*atpAGD* | *atpAGD* under control of constitutive CP41-lacLM promoter, Erm^R^ | [2] |
| pSB-T2g | *gfpmut3* under control of IPTG inducible *lacI^q^*/*P_trc_* promoter system, pMB1 replicon, Kan^R^ | [3] |
| pZA31-luc | *luc* under control of doxycyclin inducible *P_LtetO-1_* promoter system, p15A replicon, Cm^R^ | [4] |
| pSB38.2 | *gfpmut3* under control of m-toluate inducible *xylS*/*P_m_* promoter system, RK2 replicon, Amp^R^ | [5] |
| pSB74.5 | *atpAGD* under control of IPTG inducible *lacI^q^*/*P_trc_* promoter system, p15A replicon, Amp^R^ | This study |
| pSB76.2 | pSB74.5 without *atpAGD* (empty control plasmid), p15A replicon, Amp^R^ | This study |
| **Primer** | **Sequence (5’ → 3’)** | |
| atpAGD_mono_fw | CATGAACATATGCAACTGAATTCCACCGAAATC | |
| atpAGD_mono_rv | CTAGAGGATCCTTAAAGTTTTTTGGCTTTTTCC | |
| p15A_SpeI_fw | GATCACTAGTAACAACTTATATCGTATG | |
| p15A_AscI_rv | CTGAGGCGCGCCGGATATATTCCGCTTCCTCGCTC | |
| pSB_MT_backbone_fw | CAACAAAGCTCTCATCAACCG | |
| pSB_MT_backbone_rv | CTGCAGGTCGACGGATCTTTTC | |
| Amp_Gibson_fw | AAAGATCCGTCGACCTGCAGAATGTGCGCGGAACCCCTATTTG | |
| Amp_Gibson_rv | GGTTGATGAGAGCTTTGTTGCGTACTATCAACAGGTTGAAC | |

**References:**

1. Erian AM, Gibisch M, Pflügl S. Engineered *E. coli* W enables efficient 2,3-butanediol production from glucose and sugar beet molasses using defined minimal medium as economic basis. Microb Cell Fact. 2018;17:190.

2. Koebmann BJ, Westerhoff HV, Snoep JL, Nilsson D, Jensen PR. The glycolytic flux in *Escherichia coli* is controlled by the demand for ATP. J Bacteriol. 2002;184:3909-16.

3. Balzer S, Kucharova V, Megerle J, Lale R, Brautaset T, Valla S. A comparative analysis of the properties of regulated promoter systems commonly used for recombinant gene expression in *Escherichia coli*. Microb Cell Fact. 2013;12:26.

4. Lutz R, Bujard H. Independent and tight regulation of transcriptional units in *Escherichia coli* via the LacR/O, the TetR/O and AraC/I_1_-I_2_ regulatory elements. Nucleic Acids Res. 1997;25:1203-10.

5. Boecker S, Zahoor A, Schramm T, Link H, Klamt S. Broadening the Scope of Enforced ATP Wasting as a Tool for Metabolic Engineering in *Escherichia coli*. Biotechnol J. 2019;14:1800438.
